# Consequences of the cost of living: is variation in metabolic rate evolutionarily significant?

**DOI:** 10.1098/rstb.2022.0498

**Published:** 2024-02-26

**Authors:** Amanda K. Pettersen, Neil B. Metcalfe

**Affiliations:** ^1^ School of Biodiversity, One Health and Veterinary Medicine, University of Glasgow, Glasgow, G12 8QQ, UK; ^2^ School of Life and Environmental Sciences, The University of Sydney, Sydney, Australia

**Keywords:** metabolism, evolution, selection, heritability, pace of life

## 1. Introduction

The optimal animal, born with some amount of energy, proceeds through its life gaining and expending energy according to some schedule that maximises its total reproductive output (Schoener 1971 [1, p. 375])

Metabolic rate reflects energy turnover that fuels essential biological processes common to all life. This fundamental measure can scale from cells to ecosystems, providing a rate at which oxygen and resources are consumed from the environment [2]. From an evolutionary perspective, measures of metabolic rate inform theoretical predictions for how metabolic rates vary at both the scale upon which selection acts—individual variation—and the level at which adaptive responses can be identified—populations. At the level of individuals, this metric for the cost of living is ubiquitous to all living organisms, hence metabolism is perhaps the most well-studied physiological rate. Metabolic rate can be referred to as a ‘hub trait’ [3]—it can be affected by cell size, activity level and life stage, as well as biotic and abiotic factors. However, it is important to recognize that there is not a single metabolic rate—over time, and depending on the animal's activity level, its metabolism will range between a minimal or resting level (i.e. basal or standard metabolic rate) and the upper limit (maximum or summit metabolic rate), the difference between them being termed the aerobic scope; it is therefore important to clarify which metabolic rate is being considered (see [4] for further discussion and definitions).

**Figure 1.**
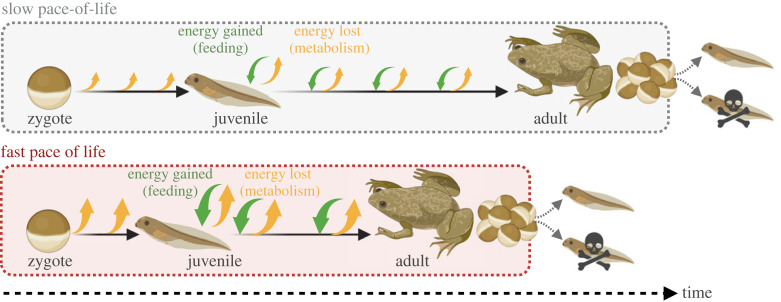
Variation in metabolic rates and the pace-of-life. Theory and some empirical evidence suggest that higher resting metabolic rates are correlated with a faster pace-of-life, such that high metabolic phenotype individuals develop and grow faster, reproduce sooner, and have shorter lifespans than individuals with low metabolic rates. Small/large orange arrows represent low/high metabolic rates, green arrows represent food intake. Dotted areas represent lifespan. It is currently unclear whether metabolic rates are a driver or product of a fast or slow pace-of-life. The potential fitness consequences (indicated by surviving offspring on the right-hand side of the figure) are likely to be context dependent.

Metabolic rates are also often but not always correlated with physiological, life-history and behavioural traits—sometimes grouped together into a ‘pace-of-life’ spectrum ([5–7]; figure 1). For example, higher resting metabolic rates have been associated with faster developmental and growth rates, earlier onset of reproduction and shorter lifespan [8]. Individuals with relatively higher metabolism are also sometimes found to be bolder, more aggressive and more able to compete for resources than individuals with lower metabolic rates [9]. On the other hand, possessing a higher minimal metabolic rate also potentially indicates a higher cost of living (and thereby lower energetic efficiency) [10]. What is less clear, is whether heritable variation in metabolic rate directly affects fitness, so that metabolic rate is able to evolve so as to maximize fitness in a given local environment. By scaling up from within- and among-individual variation to population-level processes, we can start to explain why variation in metabolic rates exists. More importantly, the current pace of environmental change makes it especially relevant to understand whether metabolic rates are able to evolve to match an ever-changing environment.

Despite its intense research focus over the last century, there is an intriguing amount of unexplained within-species variation observed in metabolic rates. Resting metabolism can vary up to threefold among individuals of equivalent body mass, age, sex, body temperature and activity level [10]; there is similar intraspecific variation in maximal metabolic rate and aerobic scope [11]. However, a potential implication of variation in these various types of metabolic rates is that they directly or indirectly influence survival and reproduction and may therefore evolve in response to selection. The purpose of this special issue ‘The evolutionary significance of variation in metabolic rates' is to provide physiologists and ecologists with lines of evidence (or in places highlight the lack of evidence) that variation in metabolic rate is important in and of itself from an evolutionary perspective. This issue covers multiple approaches—spanning scales of biological organization (genes, cells, individuals, populations and communities) and study systems (aquatic and terrestrial, ectotherms and endotherms)—to provide the latest analytical, theoretical— and methodological insights into our understanding of the potential consequences of the cost of living.

## 2. Mechanistic versus phenomenological explanations for metabolic rate variation

Physiologists have long been fascinated with understanding variation in metabolic rate—the so-called ‘fire of life’ [12]. Our current understanding of variation in metabolic rate (both minimal and maximal) is grounded in traditional respirometry measures that span centuries of research [12]. Some of the earliest tests of theoretical among-species scaling relationships [13] between metabolic rate and body mass arose through observations within species [14] (cited in [15,16]). This then later led to the foundations of metabolic theory—a series of predictions about the mechanisms that explain the less-than-proportional increase of resting metabolism with body mass that is seen when comparing across species (i.e. hypometric scaling, for a recent review, see [17]). These proposed mechanisms, often derived from first principles, are based on proximate functional causes which produce single scaling exponents through biophysical models (e.g. the metabolic theory of ecology [18] and dynamic energy budget theory [19]). Yet, there remains little support for a single metabolic scaling exponent [20,21], which may be explained by statistical artefact [22]; nor is there support for a single mechanism, since they often seem to be taxon specific. The limitations of existing metabolic theories to provide general explanations for variation in resting metabolic rate have already been recently covered (e.g. [23]). Importantly, general mechanistic approaches ignore the fascinating variation that exists after accounting for one or two factors (e.g. mass, temperature, oxygen). Rather than a product of any single mechanism, this variation in metabolic rates probably arises from evolution in response to a range of interactive intrinsic and extrinsic factors (for a review, see [3]).

An alternative approach to enhance our understanding of metabolic rate variation among species is to investigate its fitness consequences within species. This approach, which has already helped to explain allometric metabolic scaling [24,25], does not diminish the role of physical constraints that maintain metabolism within physically possible ranges [26]. Rather, studying the ultimate causes for variation in metabolism provides a means to examine potentially evolutionarily significant variation. If variation in metabolic rate is more than just a product of physical constraints imposed by the environment, then it probably has the capacity to evolve as a result of the evolutionary processes of genetic drift and selection. If either random mutation or plastic responses of metabolic traits can be inherited, then this sets the course for understanding the evolutionary trajectory of metabolic rates in populations over time [27]. In recent years, evolutionary theory has been used to parameterize components of the breeder's equation (i.e. selection [28,29] and heritability [30,31]) in the context of metabolic rates. This approach has the potential to improve our understanding of variation in metabolism measured at the whole-animal level and at other levels covered in this issue, including tissue-specific [32,33], cellular [34] and subcellular [33] metabolic activity. Genomic advances have allowed for studies that pinpoint genes involved in producing metabolic rate variation, as highlighted by Prokkola *et al*. [32]. These studies all link metabolic rate variation with some aspect of individual physiological function, and therefore potentially fitness.

Taking a phenomenological approach has shown that it is not necessary to invoke a constraint to explain variation in metabolic rate [25]. However, this approach does not rule out potential constraint(s). Indeed mechanistic (constraint) and phenomenological (optimization) views can provide potentially complementary ideas about metabolic variation despite recent debate [22,35]. It is possible that physical constraints set the outer limits to metabolism (i.e. the minimal and maximal levels) for a given body mass, while evolutionary processes (drift and selection) explain some of the variation in the operating levels of metabolism (e.g. an animal's daily energy expenditure). Selection may also act upon metabolic efficiency, measurable as the amount of ATP that is produced per unit of oxygen or substrate that is consumed by the mitochondria [36]. Both proximate and ultimate mechanisms are likely to interact, and future integration of these approaches is important. In the meantime, it is crucial that proponents of particular theories clearly state and rigorously test the assumptions of their own theory to drive forward our understanding of the causes and consequences of metabolic rate variation.

## 3. Repeatability and phenotypic plasticity of metabolic rates

**Figure 2.**
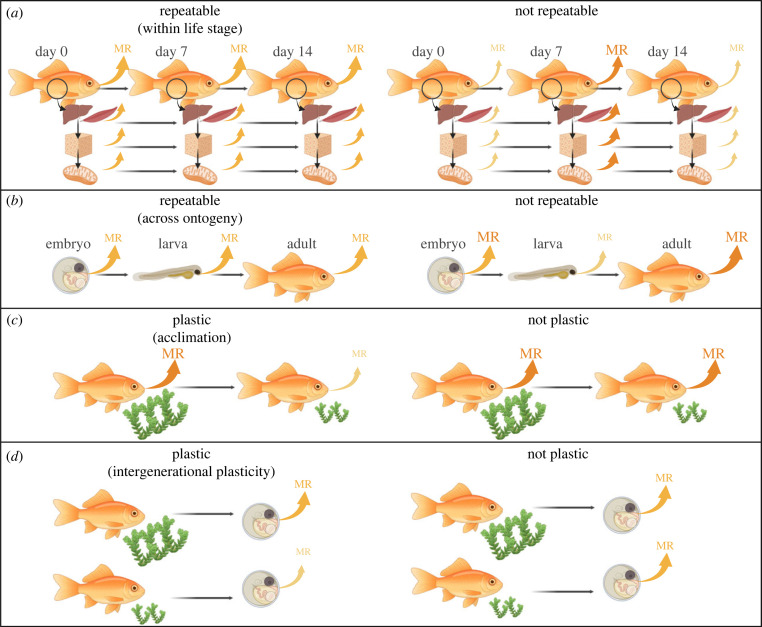
A visual representation for the difference between repeatable and non-repeatable metabolic rates (MR) (*a,b*) and different forms of metabolic rate phenotypic plasticity (*c,d*). (*a*) When metabolic rates are repeatable within a life stage (e.g. adult life stage), they will show consistency across technical replicates (measured at short time intervals). Repeated samples are often measured at the whole-organism level; however, recent technological advances are facilitating repeated non-destructive measures at the organ, tissue and subcellular level. (*b*) Metabolic rates are often less repeatable over longer time scales, such as among life stages (e.g. embryo, larval and adult life stages). When metabolic rates are repeatable across ontogeny, they should stay consistent at these life stages, such that an individual maintains a relatively low or high metabolic rate phenotype. (*c*) Individuals can show reversible plasticity (i.e. acclimation) when exposed to environmental change. For example, when food availability decreases, individuals can suppress their resting metabolic rates, potentially to conserve energy reserves. (*d*) Parents can also modify the metabolic phenotypes of their offspring in response to their environment, known as inter-generational plasticity or trans-generational plasticity (when measured across two or multiple generations, respectively). For example, when exposed to low food availability, parents can produce offspring with lower resting metabolic rates, so helping them to conserve energy reserves.

Despite being labile traits that change across environments, both minimal and maximal metabolic rates are often repeatable within an individual. Technical replicates show metabolic rates (across scales from mitochondria to whole animal) are similar across short time scales ([33]; figure 2*a*). Across ontogeny, individuals with low and high minimal metabolic rates early in life often maintain relatively low and high metabolism later in life, respectively ([37–39]; figure 2*b*). However, repeatability generally decreases over time, particularly under variable or natural conditions [40–42]. Different metabolic traits vary in their repeatability—Thoral *et al*. [33] show that basal mitochondrial respiration and net phosphorylation efficiency of mitochondria were repeatable over measurements 14 days apart, whereas there was a lower repeatability for individual oxygen consumption (standard and maximal metabolic) rates. It is therefore important to consider that metabolic rate is not a single trait, but rather multiple traits with differing degrees of flexibility, that can be measured across scales (e.g. subcellular, cellular, whole animal), activity levels (e.g. basal, standard, maximal), and ontogeny (e.g. embryo, juvenile, adult) [43,44]. Interactions between these levels may be complex. For example, Privalova *et al*. [34] show that the predicted relationship between cell size and metabolic performance, measured as whole-animal thermal tolerance, varies between the sexes and with mutations that influence cell cycle control pathways.

Quantifying within- and among- individual temporal variation in metabolic rates can reveal important insights into the evolutionary significance of variation in metabolism. For example, a study on red squirrels with varying activity levels across seasons revealed fitness advantages for possessing a high resting metabolic rate during autumn and a low resting metabolic rate during winter [44]. Repeatability of residual resting metabolic rate was significant during initial measures in autumn, but decreased when comparing across seasons. Repeatability among individuals may also be context dependent (such as under stable versus fluctuating conditions). For example, Norin *et al*. [45] show strong among-individual variation in the thermal plasticity of resting to maximal metabolic rates, with higher repeatability of both resting and maximal metabolism at warm compared with cold temperatures. Repeatability is an important measure to consider as it often sets the upper limit to heritability [46], since among-individual trait variation is necessary for adaptive phenotypic evolution [47]. Whether the repeatability of metabolism is owing to underlying genetic constraints, or whether high repeatability serves a fitness advantage, is likely to be context-dependent and requires further investigation.

Individuals can alter their metabolic rates (and even their offspring's metabolic rates) in response to internal or external environmental cues via phenotypic plasticity [40]. For example, individuals can suppress their resting metabolic rates in response to low feeding regimes [48] or increase this metabolism when exposed to cold temperatures—a form of plasticity known as acclimation ([49]; figure 2*c*). Environments experienced during early development can also modify metabolic rate expressed later in life via developmental plasticity. It is therefore important to consider not only individual variation in metabolic rates, but also individual variation in phenotypic plasticity (i.e. reaction norm slopes). Two papers in this issue discuss whether among-individual variation in metabolic responses to ambient temperature may confer fitness benefits under climate change, particularly for ectotherms. Gvoždík [50] shows consistent inter-individual variation in both the elevation and slope of the metabolic response to temperature in ectotherms, providing evidence that the thermal plasticity of metabolic rates can vary among individuals [45,50]. Moreover, Norin *et al.* [45] show that individuals with the highest metabolic and growth rates also possess the highest thermal sensitivity (i.e. steepest thermal reaction norms) in these traits. Yet the opposite trends have also been found [51], suggesting that the relationship between thermal reaction norms intercepts and slopes of individual metabolic rates depends on taxa, thermal history, and the method used to measure them (e.g. rate of temperature change, warm versus cooling) [52].

Parental effects can also serve as a significant source of variation in metabolic rates [10]. Evidence shows that parents can modify the metabolic phenotypes of their offspring across single and multiple generations, via inter- and trans-generational phenotypic plasticity, respectively ([53]; figure 2*d*). Females exposed to environmental stressors have been shown to increase the metabolic rates of their offspring [54,55], such as via elevating egg yolk hormones [56–59]. Pettersen *et al*. [60] show that an intergenerational decrease in offspring standard metabolic rate in response to warmer temperature and low food environments aligns with the direction of selection on metabolic rates (whereby fitness is measured as offspring survival), but only when parent and offspring environments match. Further measures are needed to show the conditions under which inter- and trans-generational effects on offspring metabolism are adaptive, such that they align with selection on metabolic rates. While phenotypic plasticity in metabolic traits is generally assumed to enhance offspring fitness, shifts in parental investment can also be costly for offspring [61]. Alternatively, its effects may be neutral, serving no fitness benefit or the level of plasticity in metabolic rate may be constrained by plasticity in other traits [45]. Further research quantifying the amount of among-individual variation in metabolism explained by plastic versus genetic sources is needed to begin to understand the heritability and therefore evolution of metabolic flexibility both within and across generations. Given the unprecedented rate and scale of human-mediated environmental change, the relative role of phenotypic plasticity in metabolic rates facilitating adaptation is an important challenge to address.

## 4. Is variation in metabolic rate adaptive?

…I am convinced that Natural Selection has been the most important, but not the exclusive, means of modification (Darwin 1872 [62, p. 22])

A key question that has emerged from measures of metabolic rates, is whether variation in metabolism is related to fitness [27]. Lande & Arnold's seminal paper [28] provided a multiple regression approach to estimating the form and strength of selection acting on correlated traits (see [63] for a detailed discussion). While this statistical framework was proposed over 40 years ago, it has rarely been implemented to analyse selection on metabolic rates (for a list of studies see table 1 in [27]). Furthermore, most studies do not measure actual fitness (the number of surviving offspring produced by an individual after a single generation), but instead more logistically feasible proxies of fitness, such as survival and fecundity (i.e. lifetime reproductive success). On this basis, a growing number of studies have shown evidence for selection on metabolic rates, including under natural conditions (summarized in [64]). For a description of the different forms of selection, see [63].

A major limitation of our understanding of selection on metabolic rates is that even tractable proximate measures of fitness (e.g. lifetime reproductive output and survival) are notoriously difficult to quantify under field conditions for many species. Most studies use some measure of performance instead (e.g. growth, activity level, aerobic scope) under laboratory conditions, to infer the fitness implications of metabolic rate variation in nature. Some key issues here are that (i) fitness or performance measured in the laboratory may not reflect those in the field, and (ii) performance traits may trade off with actual fitness. Indeed, a recent meta-analysis found that while resting metabolic rate has a positive relationship with a range of performance traits, there is no consistent relationship with either reproduction or survival traits [64]. Arnold *et al*. [64] conclude that further measures of lifetime reproductive success are crucial for discerning the conditions under which metabolic rates affect fitness (e.g. [8]). We therefore need longitudinal studies that capture among-individual variation in metabolic rates and lifetime reproductive output. The application of selection analysis can then be used to produce selection estimates that are comparable across metabolic traits, study systems and environments, to begin elucidating the generality of adaptive metabolic rate variation.

Evolutionary theory predicts that over time, persistent selection on a trait should deplete genetic, and therefore phenotypic, variance [65]. Hence, if metabolic rates possess heritable variation that is under selection, then it raises a paradox about how variation in metabolic phenotypes is maintained. One potential explanation is that the fitness benefits of a fast or slow metabolism change across spatiotemporal changes in the environment, also known as context-dependent selection [10,66,67]. Previous work has shown that a high resting metabolic rate (associated with a fast pace of life) is beneficial in cool [68] or high competition [66] environments, but can be either beneficial [69] or costly [70] in high predation environments. A high resting metabolic rate in juvenile Atlantic salmon is beneficial in high resource environments since it is linked to faster processing of food relative to juveniles with a lower metabolic rate [71]. Another mechanism for maintaining metabolic variation may be that selection on metabolic rates during one life stage may oppose selection on metabolic rates at another life stage, i.e. negative correlational selection on metabolic rates. For example, Pettersen *et al*. [8] found negative correlational selection on metabolic rates ('early’ and ‘late') that were measured only 24 h apart, whereby lifetime reproductive output was maximized when individuals possessed either a low MR_early_ and high MR_late_ or vice versa. In this study, individuals with consistently low or high metabolic rates showed the lowest reproductive output.

Another limitation when inferring the adaptive potential of metabolism is our understanding of whether metabolic rate is indeed the direct target of selection. Selection on one phenotype can produce indirect effects on the distribution of correlated phenotypes, often complicating the interpretation of selection analysis [28]. Metabolic rates are generally correlated with other life-history and physiological phenotypes (figure 1), which raises the question of whether metabolic rate is a driver or by-product of the pace-of-life. Furthermore, the plasticity of metabolic rates may also covary with plasticity in other key physiological and behavioural traits (e.g. rates of movement; [45]), making it challenging to resolve how these traits are likely to coevolve. If there is a sufficiently large sample size the responses of different phenotypes to selection can be partitioned using multivariate statistics into direct versus correlated effects, to determine which phenotypes are the focus of direct selection [28,72] and so separate direct selection on metabolic rates from indirect selection on other correlated traits (e.g. development time, growth, age at onset of reproduction; table 1). This can be applied when there are weak correlations among traits, i.e. less than 0.5 [77], or even less than 0.28 [78]. With regards to body mass, which is often highly correlated with metabolic rate, Cameron & Marshall [63] discuss in this issue the potential pitfalls of including mass or mass-specific metabolic rates when estimating selection on metabolic rates. They instead recommend using mass-independent metabolic rate to estimate selection on metabolic rates that are independent of body size (table 1).

**Table 1.** Current issues limiting our understanding of the evolutionary significance of variation in metabolic rates, and a summary of potential solutions. (For more details, refer to the relevant references provided.)

issue	summary of potential solution	relevant references
producing standardized estimates of selection on metabolic rates	use a classic multiple regression framework to provide standardized (and therefore directly comparable) selection coefficient estimates for viability selection (using survival data), fertility selection (using survival to reproduction data), fecundity selection (using reproductive output data)	[27–29]
identifying whether metabolic rates are the target of selection (versus under indirect selection on another weakly correlated trait)	incorporate large sample sizes into the experimental design—even if traits are highly correlated, it should be possible to tease apart the effects of different traits on fitness (unless traits and metabolic rates are perfectly correlated). Alternatively, structural equation modelling (path analysis) may be used to tease apart direct and indirect effects	[28,73]
producing comparable estimates of heritability of metabolic rates	breeding design, artificial selection or experimental evolution experiments	Box 3 in [27]
isolating the effects of a single factor on the evolution of metabolic rate when comparing populations	experimental work manipulating those variables, e.g. common garden and reciprocal transplant	[74]
explaining clines in metabolic rate in nature	two complementary approaches: (i) artificial selection to generate replicate lines that differ in metabolic rate, to assess relative fitness across treatments representing environmental clines; (ii) laboratory natural selection to observe how metabolic rates evolve under different environments while keeping generation times similar across treatments, and allowing for natural variation in population size	[74]
accounting for ‘group phenotypic composition’ as a potential driver of metabolic rate variation	artificial selection approach that modifies the composition of metabolic phenotypes within groups, to observe evolutionary trajectories in metabolism	[75,76]
avoiding collinearity between mass and metabolism when estimating selection on metabolic rates	use mass-independent metabolic rate to estimate selection on metabolic rates that are independent of body size	[63]

**Figure 3.**
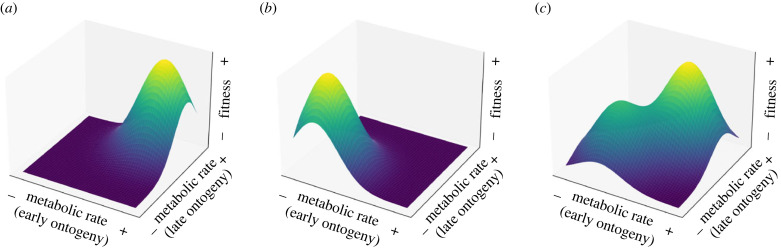
Hypothetical fitness landscapes for metabolic rate measured early and late in ontogeny and across different environments. Colour scale from blue to yellow indicates low to high fitness, respectively. In high resource environments, there may be (*a*) positive correlational selection for high metabolic rates in both early and late ontogeny (alternatively selection may be relaxed such that there is no variance in fitness across the available phenotypic variation, and therefore a flat landscape surface). (*b*) When resources are low however, there may be positive correlational selection for consistently low metabolic rates throughout ontogeny, as reflected by the shape of the fitness landscape. (*c*) Alternatively, the fitness landscape may be more complex, containing multiple optima, such as a local optimum and global optimum. For example, if resource availability changes across ontogeny, it is somewhat beneficial to have intermediate metabolic rates through life (local optima) but most beneficial to have a metabolic rate that changes to match resource availability (global optima). Assuming metabolic rates have a genetic basis, allele frequencies that underlie metabolic rate phenotypic variance will evolve via natural selection to reach a local adaptive peak that may not be the highest adaptive peak on the landscape.

The complex nature of the fitness landscape may help to explain why selection on metabolic rates is often undetected or inconsistent [64]. In cases where selection on metabolic rates is observed, the consequences of a high or low metabolic rate are likely to be highly dependent on both the energy requirements of the specific life stage being measured and the environment the organism is experiencing at the time. Selection ‘at all times acts to increase the fitness of a species to live under the conditions that existed an instant earlier’ [79, p. 131]. Hence, populations are continually evolving in an attempt to reach the closest adaptive peak (i.e. local optima for metabolic rate), which may depend on ontogenetic stage and may change over space and time (figure 3). Given that environments are anticipated to become more temporally variable and habitats more spatially fragmented, it may become increasingly difficult for populations to track the local optima for metabolic rates that best suit the prevailing conditions.

## 5. What evidence is there that metabolic rates can evolve?

Natural selection is not evolution (Fisher 1930 [80, p. 7])

Even if selection is operating on metabolic rates, evolutionary theory posits that we would not observe a response of populations to that selection unless the trait of interest is heritable [31]. There are a number of methods for quantifying the proportion of the total variance in metabolic rates that is attributable to differences in breeding values (i.e. genetic variance), allowing calculation of heritabilities (table 1). Overall, studies have shown evidence for significant narrow-sense heritability (additive genetic variance relative to phenotypic variance; *h*^2^) in metabolic rates, indicating that metabolic rates are free to evolve under selection [27]. However, there is a large variation in estimates of *h*^2^ (from 0 to 0.72), and *h*^2^ tends to be higher for endotherms than ectotherms, and for active relative to resting metabolic levels [27].

An alternative explanation for the maintenance of variation in metabolic rates is that it may be owing to the effects of pleiotropy [81,82]. Genetic associations between metabolic rates and other traits may alter, and potentially constrain the directional evolution of metabolic rates, even if they are shown to be heritable and under selection when measured in isolation. The phenotypic response to selection depends not only on the genetic basis of singular traits, but also the relationship among multiple traits. A multivariate approach allows for estimates of additive genetic variance of both the individual traits, as well as their additive genetic correlation [83]. Previous work has found that metabolic rates can evolve independently of morphological and behavioural traits (as evidenced by a lack of genetic correlation; [84]) but that they are constrained by body mass and locomotor activity (significant phenotypic and genetic correlations; [85]). Crespel *et al*. [86] find strong genetic correlations between metabolic and growth traits, but no genetic correlations between metabolic rate and swimming performance, or risk taking and sociability behaviour. Hence, to understand and predict the evolution of metabolic rates in response to selection, it is important to consider whether there are covariances with other correlated life-history, behavioural, and physiological traits that are also likely to be under selection and may therefore constrain or alter the evolution of metabolism [85].

Despite the potential for context-dependent selection and genetic correlations to maintain variation in metabolic rates, field and laboratory studies have demonstrated the evolution of metabolism across populations and generations, respectively. Studying differences across natural populations can help to infer how metabolic rates are likely to evolve under different environments. Common garden and reciprocal transplant studies have been used as a space-for-time substitution where populations sampled along a spatial gradient (e.g. from high to low latitudes) have been used to infer how metabolic rates might evolve under a warming climate [87,88]. Auer *et al*. found evidence for the rapid evolution of metabolic rate across populations that had evolved under varying predation levels [69]. While among-population studies can infer patterns and mechanisms of adaptation, these studies are correlational and cannot isolate drivers from other abiotic and biotic factors that also change across the same spatial scales. Hence, population comparisons, while providing important, ecologically relevant insights, cannot disentangle multiple potential underlying causes of metabolic rate variation (table 1).

Experimental evolution can be used to isolate and test the effects of a particular variable on the evolution of a phenotype [89]. These studies generally use the short generation times and tractability of model organisms such as bacteria, flies, zebrafish and rodents, quantifying how metabolic rates evolve under controlled laboratory conditions to infer evolution under different environments in nature [89–92]. For example, Alton *et al*. [93] tested the cold-adaptation hypothesis in fruit flies, where cold environments are expected to select for higher resting metabolic rates (also known as countergradient variation). They found no fitness advantage for evolving a higher metabolic rate in cold temperatures. An artificial selection experiment by Sadowska *et al*. [90] observed a correlated evolution of increased resting metabolic rate in lines selected for high maximal rates of metabolism. Wootton *et al*. [94] found that resting metabolic rates increased in the first generation of zebrafish exposed to a warm temperature, but then decreased after six generations, such that both cool- and warm-reared lines had the same metabolic rate. Alton *et al*. [74] conducted an experimental evolution study to separate the evolutionary responses of metabolic rates to temperature and nutrition. While they found no effect of nutrition (including its interaction with temperature) on the evolution of metabolic rates, they offer solutions for future studies to address this question using protocols that allow for selection on life-history strategies that are often otherwise controlled for in experimental evolution studies (table 1).

## 6. What are the broad scale implications of metabolic rate evolution?

Natural selection can drive the evolution of phenotypic traits correlated with metabolic rates, with important ecological and evolutionary consequences. While selection acts on individual metabolic phenotypes, the metabolic composition of individuals within a group can affect resource acquisition and allocation, such as via competition or facilitation, and can thus itself also act as an agent of selection [95,96]. Future studies may need to consider whether the form and strength of selection on metabolic rates probably depends on the abundance and distribution of other phenotypes in the group (for a discussion, see [63]). For example, large groups of individuals living in low food environments may have a higher survival if members of the group have similarly low resting metabolic rates, while a homogenous group of high metabolic rate individuals may be favoured in high predation environments when metabolism correlates with boldness [69,75,97]. Alternatively, heterogeneity in metabolic rates may facilitate resource partitioning. Variation in metabolic rates is therefore a product of eco-evolutionary dynamics, where abiotic (such as temperature) and biotic factors (such as competition) can drive selection and therefore the evolutionary trajectory of metabolic rates [98]. Despite its potential importance for explaining among-individual variation in metabolic rate, the role of group phenotypic composition in driving metabolic rate variation is currently underexplored. Naug [76] advocates for the use of social insects such as honeybees as an experimental tool in this context, since they can be used to create groups containing different configurations of metabolic rate individuals, so allowing elucidation of potential eco-evolutionary feedbacks as drivers of metabolic rate variation (table 1).

Metabolic rate data collected in the laboratory can help to assess a species average physiological niche and predict potential species distributions across space and time. Penn & Deutsch [99] pair biogeographic data for 25 000 marine animal species with climatological temperature and oxygen data to provide correlative patterns among traits, spatial patterns and phylogeny. They find that the minimum partial pressure of oxygen required to maintain metabolism is lower and less temperature sensitive among (sub)tropical species compared to polar species, probably owing to local adaptation to warmer temperatures and lower oxygen availability. This geographical pattern in hypoxia tolerance is partly driven by tropical species evolving lower temperature sensitivity in metabolic oxygen demand relative to high-latitude species. Penn & Deutsch provide intriguing insights suggesting that oxygen thresholds are phylogenetically conserved and may constrain the geographical ranges of marine species. An important next step is to prove a causal relationship between metabolism and fitness, under varying oxygen availability and temperature, to determine whether these environmental factors act as a selective pressure on metabolism, thus reflecting biogeographic patterns.

## 7. Future avenues of research

Despite the quantity of metabolic rate data collected over a rich history of ecophysiology in the past century, there remain many unanswered questions regarding why and how metabolic rates evolve. The implications of these knowledge gaps are particularly pertinent under the current rate and scale of global change that appears to be increasing the pace of life and the cost of living. Here we list potential key avenues of research explored in this special issue.

### (a) Quantifying selection acting on subcellular metabolic phenotypes

Studies of selection on metabolism as a measure of the cost of living generally use proxies for metabolic rate measured at the whole-organism level, such as oxygen consumption or carbon dioxide production. However, these proxies do not always predict ATP production—the expected target of selection [36]. For example, Thoral *et al*. [33] found no significant relationship between mitochondrial metabolism and whole-animal performance. Oxygen is usually not limiting in the environment; hence the rate of oxygen consumption is not likely to be under selection. Furthermore, in the absence of oxygen, it is possible to produce ATP via glycolysis [40]. An individual's physiology is a product of its cellular machinery, yet the link between whole animal respiration and its cellular processes is a relatively recent field of investigation [36]. An understanding of how ATP production is linked to individual fitness is a crucial next step in our understanding of the adaptive significance of variation in the cost of living.

### (b) Within-individual variation in metabolic rates

Ongoing technological advances are increasing both the throughput and precision of metabolic rate measurements, and providing previously unexplored insights into metabolic rate variation [4]. Thoral *et al*. [33] provide a new method for repeated non-destructive sampling of mitochondrial function in individuals through time. To reduce measurement error associated with single measures of individual metabolic rates, Cameron & Marshall [63] recommend repeated (technical) replicates of metabolic rate over short intervals to incorporate within-individual variation (table 1). Innovations in high-throughput phenotyping are making replication increasingly feasible, providing a promising outlook for the field. The increasing capacity to measure respiration in multiple individuals simultaneously will enable biologists to quantify how selection on metabolic rates changes across ontogeny (given that metabolic rate is not a single trait).

### (c) Considering how mass as a covariate influences estimates of selection on metabolic rates

With a growing number of studies integrating measures of metabolic rates with microevolutionary theory for the first time, there are potential pitfalls that can be avoided. For example, including highly correlated independent variables can yield inaccurate estimates of selection (see §4, is variation in metabolic rate adaptive?). Fortunately, continual advances in analytical techniques provide the means to estimate quantitative genetics parameters that are robust such that they can be used to understand the evolution of metabolic rates. Cameron & Marshall [63] provide a useful guide for implementing selection analysis for metabolic rates.

### (d) Interplay between phenotypic plasticity and genetic variation in metabolic rates

Even after accounting for the genetic contribution to phenotypic variance, there remains a significant proportion of unexplained variation in metabolic rates that can be attributed to environmental effects or plasticity ([10], see §3, repeatability and phenotypic plasticity of metabolic rates). Recent evidence suggests that a degree of plasticity in metabolic rates may have an additive genetic component [100]. If plastic changes in metabolic rates are favoured by selection, then these responses (acclimation, developmental plasticity and intergenerational plasticity) may become fixed in populations over time [101]. An important next step is to quantify selection and heritability of plastic metabolic responses, such as through quantitative genetics, to determine whether they pose fitness benefits (i.e. are adaptive), carry fitness costs (are maladaptive) or are neutral, and their potential to evolve in response to selection.

### (e) Consideration of nonlinearity in predictions of metabolic rates

Metabolic rates often vary nonlinearly with environmental factors, yet predictions of metabolic variation generally lack consideration of nonlinear averaging, or Jensen's inequality [102,103]. Recent work has shown that the average metabolic response to variable environments cannot be estimated by taking the average of the two extremes measured. For example, under fluctuating temperatures, such as a diel temperature regime, the metabolic rate at the average temperature is higher than the metabolic rate predicted by averaging metabolic rates at the lower and upper temperature [104]. However, Jensen's inequality provides a useful framework for making quantitative predictions for metabolic responses under variable conditions [105]. Previous work has already shown that Jensen's inequality can predict an increase in metabolic rates and reduced energy efficiency, under fluctuating temperatures [106,107]. The implication of metabolic rates evolving under more variable regimes anticipated by climate change is that organisms will need to evolve a compensatory countergradient response (either via plastic or genetic means) [108]. A greater appreciation of the ubiquity of nonlinear averaging in natural conditions, and experimental work reflecting this complexity, will improve our understanding of the compensatory mechanisms organisms will require under a changing environment.

## 8. Conclusion

By considering not just proximate mechanisms for *how* rates evolve, but the phenomenological question of *why* metabolic rates evolve, we can tap into some powerful approaches for understanding the adaptive potential of metabolic rates across generations. The capacity for organisms to rapidly evolve energy expenditure is a key research priority in the Anthropocene.

## Data Availability

This article has no additional data.
